# Predictive analysis of economic and clinical outcomes in total knee arthroplasty: Identifying high‐risk patients for increased costs and length of stay

**DOI:** 10.1002/ksa.12547

**Published:** 2024-12-04

**Authors:** David Maman, Guy Liba, Michael Tobias Hirschmann, Lior Ben Zvi, Linor Fournier, Yaniv Steinfeld, Yaron Berkovich

**Affiliations:** ^1^ Israel Institute of Technology, Technion University Hospital, Rappaport Faculty of Medicine Haifa Israel; ^2^ Department of Orthopedics Carmel Medical Center Haifa Israel; ^3^ Department of Orthopaedic Surgery and Traumatology Kantonsspital Baselland Bruderholz Switzerland; ^4^ Pediatric Department Carmel Medical Center Haifa Israel

**Keywords:** hospital charges, length of stay, neural network, postoperative complications, total knee arthroplasty

## Abstract

**Purpose:**

The purpose of this study was to predict high‐risk patients who experience significant increases in hospital charges and length of stay (LOS) following specific postoperative complications.

**Methods:**

This study analyzed over two million patients from the Nationwide Inpatient Sample database undergoing elective total knee arthroplasty (TKA) for primary osteoarthritis. Baseline demographics, clinical characteristics and incidence of postoperative complications were examined. A neural network model was utilized to predict high‐risk patients who fall into the top 25% for both LOS and total hospital charges after complications such as sepsis or surgical site infection (SSI).

**Results:**

The most common complications were blood loss anaemia (14.6%), acute kidney injury (1.6%) and urinary tract infection (0.9%). Patients with complications incurred significantly higher total charges (mean $66,804) and longer LOS (mean 2.9 days) compared to those without complications (mean $58,545 and 2.1 days, respectively). The neural network model demonstrated strong predictive performance, with an area under the curve of 0.83 for the training set and 0.78 for the testing set. Key complications like sepsis and SSIs significantly impacted hospital charges and LOS. For example, a 57‐year‐old patient with diabetes and sepsis had a 100% probability of being in the top 25% for both total charges and LOS.

**Conclusion:**

Postoperative complications in TKA patients significantly increase hospital charges and LOS. The neural network model effectively predicted high‐risk patients after specific complications occurred, offering a potential tool for improving patient management and resource allocation.

**Levels of Evidence:**

Level III.

**Abbreviations**
AKI: acute kidney injury
DVT: deep vein thrombosis
ICD‐10: International Classification of Diseases, 10th Revision
LOS: length of stay
ML: machine learning
NIS: Nationwide Inpatient Sample
PE: pulmonary embolism
SSI: surgical site infection
TKA: total knee arthroplasty
UTI: urinary tract infection

## INTRODUCTION

Total knee arthroplasty (TKA) is increasingly performed worldwide, primarily due to population ageing and rising osteoarthritis prevalence, offering significant pain relief and functional recovery benefits to advanced knee arthritis patients [3, 6, 7, 13, 14, 17, 19, 23, 41]. However, despite these improvements, postoperative complications remain a persistent challenge. Complications such as infection, venous thromboembolism and renal failure can significantly extend hospital stays, increase healthcare costs and negatively impact recovery [1, 2, 5, 12, 15, 18, 19, 31, 36].

While many studies have examined the occurrence and impact of specific complications, fewer have comprehensively analyzed these outcomes on a large scale, especially in relation to the economic burden they pose. Current literature lacks a unified model that predicts not only the likelihood of these complications but also their combined effect on hospital charges and length of stay (LOS) [4, 8, 12, 13, 14, 30]. Moreover, advances in machine learning (ML) and artificial intelligence (AI) have yet to be fully leveraged to address this gap. Recent research has shown that AI models can outperform traditional statistical approaches in predicting postoperative risks [13, 17, 22, 38], yet their application in the context of large, nationally representative data sets like the Nationwide Inpatient Sample (NIS) remains limited.

Thus, this study seeks to fill that gap by utilizing a neural network model to predict which patients undergoing elective TKA are most likely to experience postoperative complications that significantly elevate hospital charges and LOS. The goal is to provide actionable insights that can enhance patient selection and management, ultimately improving both clinical and economic outcomes in TKA [4, 13, 16, 17, 22, 32, 34, 42, 43].

It was hypothesized that postoperative complications in TKA patients can be predicted using a neural network model and that these complications are associated with significantly higher hospital charges and LOS, necessitating targeted interventions to mitigate their impact.

## MATERIALS AND METHODS

This study utilized a data set extracted from the NIS database [10], the largest publicly available all‐payer inpatient care database in the United States. The data set comprised a total of 2,299,979 patients who underwent elective TKA for primary osteoarthritis from 1 January 2016 to 31 December 2019. The NIS is a vital component of the Healthcare Cost and Utilization Project and includes data from a 20% stratified sample of US community hospitals. The chosen time frame was specifically selected due to the introduction of International Classification of Diseases, 10th Revision (ICD‐10) coding in 2016, which provides more precise diagnostic and procedural coding compared to the older ICD‐9 system. Additionally, data after 2019 were excluded due to potential distortions caused by the COVID‐19 pandemic, which altered healthcare delivery and patient selection. As of now, no data beyond 2021 has been included in the NIS, ensuring that this data set remains reliable and unaffected by pandemic‐related biases.

### Patient identification and exclusions

Patients undergoing elective TKA were identified using specific ICD‐10 procedure codes related to total knee replacement. Exclusions included patients with nonelective admissions, revision surgeries, bilateral knee surgeries and those who underwent robotic or navigation‐assisted TKA due to their higher cost. The exclusion of these cases was intentional to maintain a focus on the impact of complications in standard surgical methods.

### Outcome measures

The primary outcome measures included total hospital charges and LOS. Postoperative complications such as blood loss anaemia, acute kidney injury (AKI), blood transfusion, urinary tract infection (UTI), venous thromboembolism, intraoperative fracture, deep vein thrombosis (DVT), pulmonary embolism (PE), pneumonia, surgical site infection (SSI), ileus, heart failure, sepsis, acute coronary artery disease, pulmonary oedema and dehiscence were analyzed.

### Neural network model training and evaluation

To predict high‐risk patients falling into the top 25% for both LOS and total hospital charges, a neural network model was implemented using MATLAB's Classification Learner app. The data set was split into 80% for training and 20% for testing. This split is a standard practice in ML, and the choice was made to ensure sufficient data for training while retaining a robust testing set. We verified that the data were not significantly imbalanced, and early stopping was applied to reduce the risk of overfitting.

We utilized multiple hidden layers and the Adam optimizer with a binary cross‐entropy loss function. Hyperparameter tuning was conducted to optimize model performance. Various combinations of learning rates, batch sizes and hidden layers were tested using grid search. The final model was selected based on its performance on the validation set, which balanced predictive accuracy with generalization to new data. Additionally, other models such as logistic regression and decision trees were tested for comparison, but the neural network outperformed them in terms of area under the curve (AUC), making it the preferred model for final analysis.

### Statistical analyses

Statistical analyses were conducted using SPSS version 26.0. The analyses included multivariate regression modelling to determine the impact of postoperative complications on total hospital charges and LOS. A *p *< 0.05 was considered statistically significant. Comorbidities were identified and validated through a meticulous review of patient‐specific ICD‐10 codes. Microsoft Excel was utilized for data visualization to create charts and graphs illustrating the study's findings.

### Ethical aspects

The study was conducted under exempt status granted by the institutional review board, with the requirement for informed consent waived due to the de‐identified nature of the NIS data set.

## RESULTS

### Baseline demographics and clinical characteristics of TKA patients

Table 1 presents the baseline demographics and clinical characteristics of patients who underwent TKA. The data include distributions of sex, age, primary expected payer, median household income, location/teaching status and region of hospitalization. The majority of patients were female (61.7%), with Medicare being the primary expected payer (57.1%). A significant portion of patients resided in urban teaching hospitals (62.4%).

**Table 1:** Baseline demographics and clinical characteristics of TKA patients.

Characteristic	Frequency	Percent
Total patients	2,299,979	‐
Sex		
Female	1,419,045	62
Primary expected payer		
Medicare	1,312,225	57
Medicaid	98,755	4
Private (including HMO)	802,955	35
Self‐pay	11,300	1
Other	72,885	3
Median household income		
0–25th percentile	491,615	21
26th–50th percentile	604,505	26
51st–75th percentile	624,275	27
76th–100th percentile	547,815	24
Location/teaching status		
Rural	238,719	10
Urban nonteaching	626,426	27
Urban teaching	1,434,874	62
Region		
Northeast	431,560	19
Midwest	628,801	27
South	810,780	35
West	428,879	19

*Note*: This table presents the baseline demographics and clinical characteristics of patients who underwent TKA. It includes distributions of sex, primary expected payer, median household income, location/teaching status and region of hospitalization. Most patients were female, with Medicare as the primary payer, and the majority were treated at urban teaching hospitals.

Abbreviations: HMO, health maintenance organization; TKA, total knee arthroplasty.

### Clinical characteristics and comorbidities of TKA patients

Table 2 summarizes the clinical characteristics and comorbidities of the TKA patient population. The most prevalent comorbidities included hypertension (59.6%), dyslipidaemia (46.7%) and obesity (31.1%).

**Table 2:** Clinical characteristics and comorbidities of TKA patients.

Comorbidity	Frequency	Percent
Hypertension	1,370,590	59.6
Dyslipidaemia	1,073,275	46.7
Obesity	714,360	31.1
Disorders of thyroid	410,880	17.9
Diabetes mellitus	500,720	21.8
Atrial fibrillation	162,175	7.1
Chronic kidney disease	159,315	6.9
Chronic lung disease	137,395	6.0
Chronic anaemia	131,880	5.7
History of cerebrovascular accident (or TIA)	93,190	4.1
Osteoporosis	90,840	3.9
Peripheral vascular disease	33,105	1.4
Liver disease	29,240	1.3
Congestive heart failure	28,275	1.2
Neoplasms	21,650	0.9
Alcohol abuse	20,490	0.9
Parkinson disease	13,105	0.6
Neoplasms of lymphoid and hematopoietic tissue	8995	0.4

*Note*: This table summarizes the clinical characteristics and comorbidities of TKA patients. The most common comorbidities include hypertension, dyslipidaemia and obesity, reflecting the general health burden of this patient population.

Abbreviations: TKA, total knee arthroplasty; TIA, transient ischemic attack.

### Incidence and frequencies of postoperative complications

Table 3 shows the incidence and frequencies of postoperative complications among TKA patients. The most common complications were blood loss anaemia (14.6%), AKI (1.6%) and UTI (0.9%).

**Table 3:** Incidence and frequencies of postoperative complications among TKA patients.

Complication	Frequency	Percent
Blood loss anaemia	334,960	14.6
AKI	36,485	1.6
Blood transfusion	27,670	1.2
UTI	20,195	0.9
Venous thromboembolism	6745	0.3
Intraoperative fracture	5955	0.3
DVT	4950	0.2
PE	4800	0.2
Pneumonia	3875	0.2
SSI	3500	0.2
Ileus	2610	0.1
Heart failure	2550	0.1
Sepsis	1355	0.1
Acute coronary artery disease	1350	0.1
Pulmonary oedema	1280	0.1
Wound dehiscence	770	0.0

*Note*: This table details the incidence and frequencies of postoperative complications in TKA patients. The most frequent complications were blood loss anaemia, AKI and UTI.

Abbreviations: AKI, acute kidney injury; DVT, deep vein thrombosis; PE, pulmonary embolism; SSI, surgical site infection; TKA, total knee arthroplasty; UTI, urinary tract infection.

### Total charges and LOS by complication status

Table 4 shows the relationship between the presence of complications and total hospital charges as well as the LOS. Patients with complications incurred significantly higher (a *p *< 0.0001) total charges and longer hospital stays compared to those without complications. The average total charges for patients without complications were approximately $58,545, whereas patients with complications had average total charges of $66,804. Similarly, the mean LOS was 2.1 days for patients without complications and 2.9 days for those with complications. These differences are all statistically significant with a *p *< 0.0001.

**Table 4:** Total charges and LOS by complication status.

Complication	Total charges (USD)	Mean LOS (days)	Std. deviation (charges)	Std. deviation (LOS)
Presence of complications				
No	58,546	2.1	32,996	1.5
Yes	66,804	2.9	44,783	2.2
Total	59,985	2.3	35,474	1.7
Blood loss anaemia	65,219	2.8	43,263	2.0
AKI	81,614	4.2	63,973	3.9
Blood transfusion	79,888	4.2	52,555	3.0
UTI	73,522	3.5	48,622	3.3
Venous thromboembolism	85,669	4.5	74,701	4.3
Intraoperative fracture	77,601	3.6	52,938	3.1
PE	94,169	5.9	65,522	3.7
Pneumonia	96,980	6.3	82,303	5.1
SSI	126,132	8.7	126,009	8.3
Ileus	98,307	6.5	87,816	5.7
Heart failure	104,953	6.9	88,823	5.3
Sepsis	156,707	10.1	150,628	10.1
Acute coronary artery disease	129,646	6.8	114,751	5.6
Pulmonary oedema	86,608	5.0	64,152	3.7
Wound dehiscence	79,818	4.7	52,776	6.3

*Note*: This table shows the relationship between the presence of complications and total hospital charges, as well as the LOS for TKA patients. Patients with complications incurred significantly higher total charges and longer hospital stays compared to those without complications.

Abbreviations: AKI, acute kidney injury; LOS, length of stay; PE, pulmonary embolism; SSI, surgical site infection; TKA, total knee arthroplasty; UTI, urinary tract infection.

### Impact of complications on LOS

**Figure 1:**
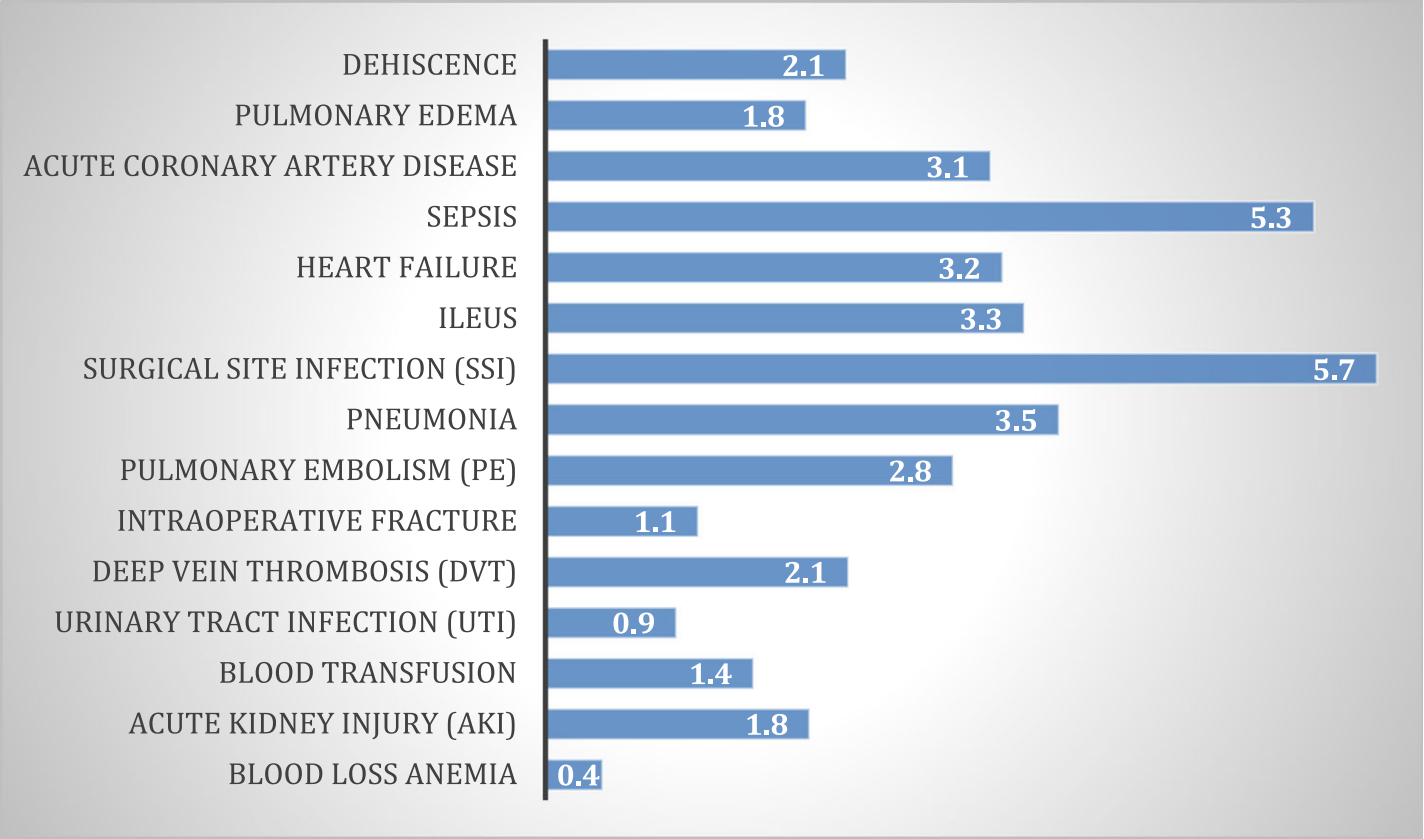
Regression coefficients for length of stay (LOS) by complication. This figure displays the regression coefficients from a multivariate analysis, showing the impact of each complication on the LOS for total knee arthroplasty patients. Sepsis and surgical site infections had the most substantial impact on LOS.

Figure 1 displays the coefficients from the multivariate regression analysis, showing the impact of each complication on LOS for TKA patients. The coefficients indicate the average additional days of hospital stay associated with each complication. A positive coefficient indicates an increase in the outcome. Sepsis and SSI had the most substantial impact on LOS, indicating their significant role in prolonging hospitalization. All the data in this figure are statistically significant with a *p *< 0.0001.

### Impact of complications on total charges

**Figure 2:**
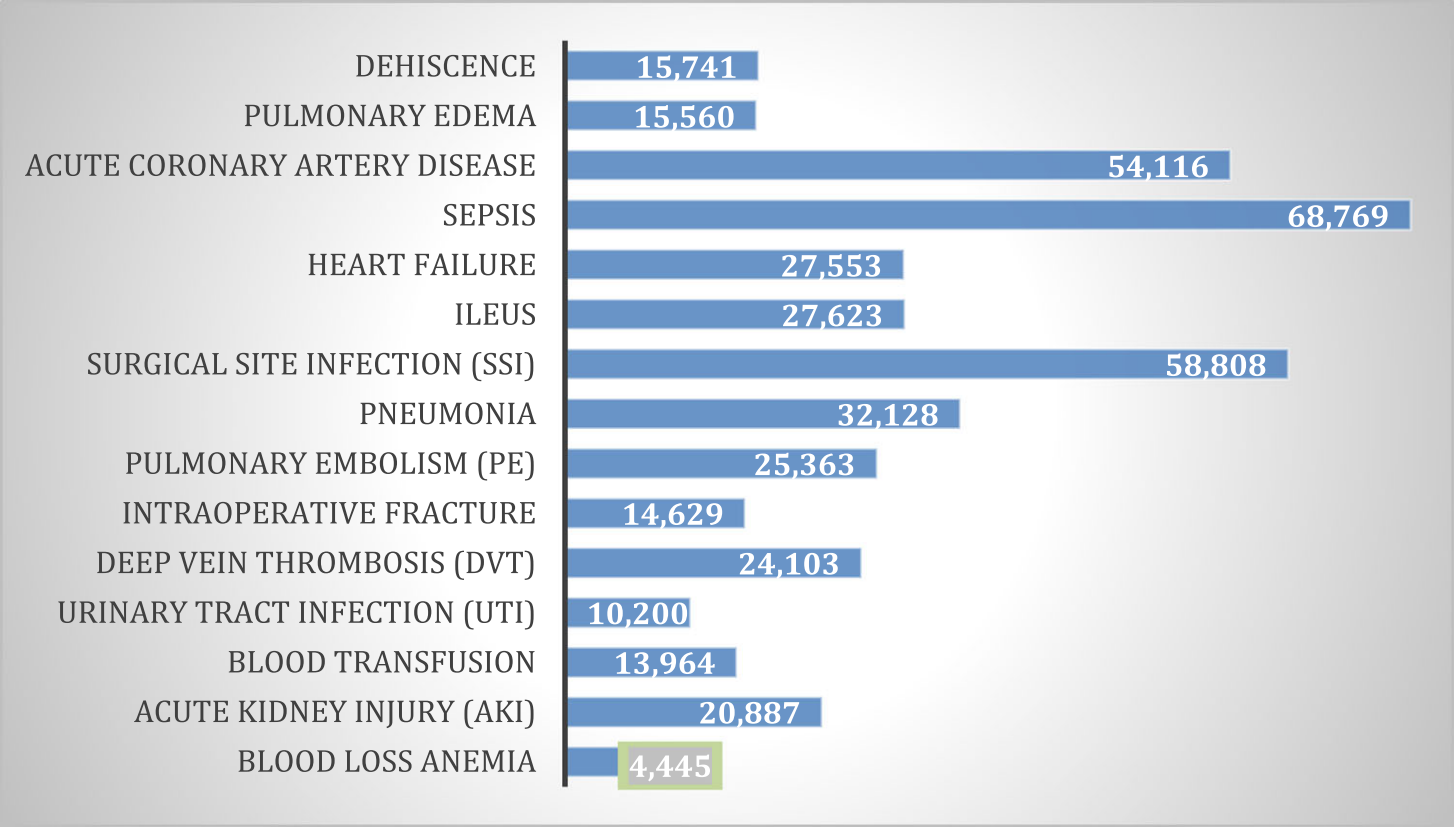
Regression coefficients for total charges by complication. This figure illustrates the coefficients from a multivariate regression analysis, indicating the impact of complications on total hospital charges for total knee arthroplasty patients. Sepsis and surgical site infections were associated with the highest additional costs.

Figure 2 shows the coefficients from the multivariate regression analysis, representing the impact of each complication on total hospital charges for TKA patients. The coefficients indicate the average additional cost in USD associated with each complication. The complications sepsis and SSI resulted in the highest additional costs, highlighting their economic impact. All the data in this figure are statistically significant with a *p *< 0.0001.

### Neural network model performance

The neural network model was designed to predict whether patients would fall to the top 25% for both LOS and total hospital charges. These predictions help identify high‐risk patients, informing resource allocation and patient management strategies.

The performance of the neural network was evaluated using several key metrics: AUC measures the model's ability to distinguish between high‐risk and low‐risk patients, with higher values indicating better discrimination. Intercept and Slope from a logistic regression model assess the calibration, with ideal values close to zero and one, respectively. The Brier score measures the accuracy of probabilistic predictions, with lower values being better.

The results, summarized in Table 5, indicate that the neural network performed well. The AUC values suggest good discriminatory power, while the intercept and slope values indicate reasonable calibration. The Brier scores reflect the accuracy of the model's probabilistic predictions. These metrics demonstrate the model's effectiveness in predicting high‐risk patients.

**Table 5:** Performance metrics of the neural network model.

Metric	Neural network training set	Neural network testing set
AUC	0.83	0.78
Intercept	−0.4	−1
Slope	1.02	0.95
Brier score	0.05	0.06

*Note*: This table presents the performance metrics of the neural network model designed to predict patients in the top 25% for both length of stay and total hospital charges. The metrics include area under the curve, intercept, slope and Brier score, highlighting the model's accuracy and predictive ability.

### Patient examples

- A 57‐year‐old patient with diabetes and no other comorbidities who had sepsis has a probability of 100% of being in the top 25% for both total hospital charges and LOS.
- An 82‐year‐old patient with no comorbidities and blood loss anaemia has a probability of 8% of being in the top 25% for both total hospital charges and LOS.
- A 66‐year‐old patient with hypertension, dyslipidaemia, obesity, thyroid disorders, diabetes mellitus, atrial fibrillation, a history of cerebrovascular accident and PE has a probability of 74% of being in the top 25% for both total hospital charges and LOS.
- A 61‐year‐old patient with no comorbidities or complications has a probability of 3% of being in the top 25% for both total hospital charges and LOS.
- A 75‐year‐old patient with chronic kidney disease, heart failure and UTI has a probability of 89% of being in the top 25% for both total hospital charges and LOS. A 45‐year‐old patient with obesity, diabetes and AKI has a 55% probability of being in the top 25% for both total hospital charges and LOS.
- A 72‐year‐old patient with hypertension, a history of MI and a DVT has a 63% probability of being in the top 25% for both total hospital charges and LOS.
- A 50‐year‐old patient with no prior medical history but who experienced an intraoperative fracture during surgery has a 48% probability of being in the top 25% for both total hospital charges and LOS.
- A 68‐year‐old patient with chronic obstructive pulmonary disease, chronic anaemia, diabetes and blood transfusion during the surgery has a 72% probability of being in the top 25% for both total hospital charges and LOS.
- A 60‐year‐old patient with atrial fibrillation and postoperative pneumonia has a 65% probability of being in the top 25% for both total hospital charges and LOS.

## DISCUSSION

The most important finding of this study was the significant increase in hospital charges and LOS associated with specific postoperative complications in patients undergoing TKA. These results underscore the substantial economic and clinical burden that complications impose on both healthcare systems and patients.

Analysis of the NIS data from 2016 to 2019 revealed that patients with complications incurred significantly higher total charges and longer hospital stays compared to those without complications. Specifically, the average total charges for patients without complications were approximately $58,545, whereas patients with complications had average total charges of $66,804. Similarly, the mean LOS was 2.1 days for patients without complications and 2.9 days for those with complications. These differences are statistically significant, with a *p *< 0.0001, highlighting the financial and clinical impact of complications in TKA.

The regression analyses further elucidate the specific impact of individual complications on total charges and LOS. Sepsis and SSI were associated with the highest increases in both total charges and LOS. Sepsis increased hospital charges by an average of $68,769 and LOS by 5.3 days, while SSI increased charges by $126,132 and LOS by 8.7 days. PE also significantly impacted hospital charges, increasing them by $58,808 and LOS by 5.7 days. Our study shows results similar to those in other studies, where sepsis and SSI significantly increased costs and extended hospital stays for patients undergoing TKA [9, 19, 31, 33, 39, 40]. These results emphasize the importance of preventing infections to reduce the substantial economic burden they impose.

A significant aspect of this study was the application of a neural network model to predict high‐risk patients, identifying those likely to fall into the top 25% for both total charges and LOS. Neural networks, as a subset of AI, excel in identifying complex patterns in large data sets, making them ideal for analyzing multifactorial outcomes like postoperative complications [4, 5, 13, 17, 21, 22, 38, 41, 42].

The neural network was trained using a comprehensive set of demographic, socioeconomic and clinical variables. This allowed for the creation of a predictive model that considers multiple factors simultaneously, offering a more nuanced prediction than traditional statistical models. The performance metrics of the neural network model, such as an AUC of 0.83 for the training set and 0.78 for the testing set, indicate strong discriminatory power in identifying high‐risk patients. The calibration metrics (Intercept and Slope) and the Brier scores further validate the accuracy of the model's predictions, making it a valuable tool for clinical decision‐making.

The neural network's ability to predict which patients are most likely to incur high hospital charges and extended LOS offers a promising avenue for improving patient management and resource allocation. By identifying high‐risk patients before surgery, healthcare providers can implement targeted interventions, optimize resource use and potentially prevent complications. These findings align with previous studies, which have demonstrated the utility of AI and ML models in predicting surgical complications, such as renal failure and postoperative blood transfusions [4, 5, 10, 17, 22, 28, 35, 37, 38, 42, 43].

Furthermore, advancements in deep learning algorithms have enhanced the predictive accuracy of AI models in orthopaedic surgery [11, 17, 20, 28, 29, 43]. This study contributes to the growing body of evidence supporting the integration of AI into clinical workflows to optimize patient outcomes. AI tools like neural networks can complement traditional risk assessments, providing a more comprehensive approach to patient selection and postoperative care.

The study has several limitations. First, it did not distinguish between different types of complications beyond their general categories, which could provide more detailed insights into their specific impacts. Second, the NIS database, while comprehensive, may contain coding errors and lacks long‐term follow‐up data [24, 25, 26, 27]. Additionally, the retrospective nature of the study limits causal inferences.

Future research should focus on the long‐term cost‐effectiveness and clinical outcomes of TKA, considering the impact of advanced surgical technologies and enhanced recovery protocols. Understanding these factors will help in making informed healthcare decisions that balance patient care improvements with financial sustainability. Further studies should also explore strategies to prevent high‐cost complications, thereby reducing the overall economic burden on healthcare systems. Additionally, refining neural network models to include more granular data on specific complications and incorporating real‐time data could further enhance predictive accuracy and clinical utility.

## CONCLUSION

This study shows that postoperative complications, particularly SSI and sepsis, significantly increase hospital charges and LOS in TKA patients. The neural network model effectively predicted high‐risk patients, demonstrating its potential to improve patient management and resource allocation. Our findings highlight the need for targeted strategies to reduce complications and optimize outcomes. Future research should focus on further refining these predictive models and exploring their long‐term impact.

## AUTHOR CONTRIBUTIONS

David Maman conducted major parts of the work, including data analysis and manuscript writing. Guy Liba assisted with manuscript writing. Michael Tobias Hirschmann provided clinical expertise. Lior Ben Zvi contributed to manuscript writing. Linor Fournier contributed to manuscript writing and data analysis. Yaniv Steinfeld contributed to revision writing. Yaron Berkovich conceived the study idea, mentored the project and supervised the work.

## CONFLICT OF INTEREST STATEMENT

The authors declare no conflict of interest.

## ETHICS STATEMENT

The authors have nothing to report.

## Data Availability

The data that support the findings of this study are available from the National Inpatient Sample (NIS). However, restrictions apply to the availability of these data, which were used under license for the current study, and so are not publicly available. Data are available from the authors upon reasonable request and with permission of the NIS.
